# Supplementary material for: Exceptional stability of a perilipin on lipid droplets depends on its polar residues, suggesting multimeric assembly
Source: eLife. 2021 Apr 15;10:e61401. doi: 10.7554/eLife.61401 (PMC8064757; doi:10.7554/eLife.61401)
Supplement: Supplementary file 2. — Table summarizes input values and calculations for five experimental conditions (A-E) used to standardize the fluorescence of Alexa488 labeled Plin4 12mer on lipid surface, using bead-supported diphytanoyl bilayers as standards. [file elife-61401-supp2.docx]

**Supplementary file 2. Calculation of Plin4 12mer density on oil, related to Figure 8 B-D.** Table summarizes input values and calculations for 5 experimental conditions (A-E) used to standardize the fluorescence of Alexa488 labelled Plin4 12mer on lipid surface, using bead-supported diphytanoyl bilayers as standards.

| Experiment | A | B | C | D | E |
| --- | --- | --- | --- | --- | --- |
| Reaction volume (*V*) | 30 μl | 30 μl | 30 μl | 30 μl | 30 μl |
| [Plin4 12mer] (nM) ^(1)^ | 80 (0.25) | 80 (0.25) | 80 (0.25) | 80 (0.25) | 32 (0.25) |
| Plin4*:Plin4 ratio (*r*) ^(2)^ | 1 : 20 | 1 : 5 | 1 : 5 | 1 : 5 | 1 : 5 |
| Bead Number (*N*) ^(3)^ | 1.7 x 10^6^ (0.2) | 1.7 x 10^6^ (0.2) | 3.4 x 10^6^ (0.2) | 5.1 x 10^6^ (0.2) | 1.7 x 10^6^ (0.2) |
| Fraction of Plin4 bound to beads (*f*) ^(4)^ | 0.6 (0.17) | 0.6 (0.17) | 0.75 (0.13) | n.d. | 0.6 (0.33) |
| Fl. Intensity on beads (A.U.) (*F_Plin4_ _bead_*) ^(5)^ | 225 (0.1) | 1100 (0.23) | 350 (0.21) | 200 (0.5) | 500 (0.15) |
| Fl. Intensity on oil (*F_Plin4_ _oil_*) ^(6)^ | 2350 (0.4) | 2350 (0.4) | 2350 (0.4) | 2350 (0.4) | 2350 (0.4) |
| Surface density of Plin4 12mer (nm^-2^) ^(7)^ | 0.067 (0.55) | 0.054 (0.59) | 0.108 (0.57) | n.d. | 0.048  (0.73) |
| Molecular area of Plin4 12mer (nm^2^) | 15 +/- 8 | 18.5 +/- 11 | 9.3 +/- 5.3 | n.d. | 21 +/- 15 |

(1) Concentration of Plin4 12mer was determined from SDS PAGE stained with Coomassie blue or SyprOrange after centrifugation at 100 000 g of thawed protein stock. Values are mean +/- relative SD (in parentheses) from 3 independent measurements.

(2) Unlabelled Plin4 12mer (Plin4) was mixed with Alexa488-labelled Plin4 12mer (Plin4*) at indicated molar ratios.

(3) Bilayer-supported beads were prepared as described, diluted in a 30x stock solution and counted under the microscope before adding it to the final reaction. The number of beads in the final reaction was determined from the concentration in stock solution and the dilution factor.

Total bead surface area was calculated by multiplying the number of beads in the reaction with surface area of an individual bead (r = 5 x 10^3^ nm).

(4) Fraction of Plin4 12mer bound to beads was determined in a fluorimeter by measuring the fluorescence of protein solution before and after incubation with beads for 15 min at RT. Values are mean +/- relative SD (in parentheses) from 3 independent measurements for each condition; n.d., not determined.

(5) Fluorescence intensity of Plin4 12mer-A488 on the surface of beads from the experiment shown in Fig. 8D. Values are mean +/- relative SD (in parentheses) from 20 beads per condition.

(6) Fluorescence intensity of Plin4 12mer-A488 on the surface of oil was determined in two independent experiments shown in Fig. 8B from 75 particles in total.

(7) Surface area density of Plin4 12mer on oil was calculated using the formula:

$Surface Density =N_{av}\left[ Plin4 12 mer \right]\times f_{bound}\times\frac{1}{r}\times\frac{{Vol}_{reaction}}{\left( N_{beads}\times bead area \right)}\times\frac{F_{Plin4 oil}}{F_{Plin4 beads}}$

where *N_av_* is the Avogadro Number

Relative error given in parenthesis was calculated from the square root of the sum of all input relative error.
