# Supplementary material for: Exceptional stability of a perilipin on lipid droplets depends on its polar residues, suggesting multimeric assembly
Source: eLife. 2021 Apr 15;10:e61401. doi: 10.7554/eLife.61401 (PMC8064757; doi:10.7554/eLife.61401)
Supplement: Transparent reporting form [file elife-61401-transrepform.docx]

***eLife’s* transparent reporting form**

We encourage authors to provide detailed information *within their submission* to facilitate the interpretation and replication of experiments. Authors can upload supporting documentation to indicate the use of appropriate reporting guidelines for health-related research (see [EQUATOR Network](http://www.equator-network.org/%20)), life science research (see the [BioSharing Information Resource](https://biosharing.org/)), or the [ARRIVE guidelines](http://www.plosbiology.org/article/info:doi/10.1371/journal.pbio.1000412) for reporting work involving animal research. Where applicable, authors should refer to any relevant reporting standards documents in this form.

If you have any questions, please consult our Journal Policies and/or contact us: [editorial@elifesciences.org](mailto:editorial@elifesciences.org).

**Sample-size estimation**

- You should state whether an appropriate sample size was computed when the study was being designed
- You should state the statistical method of sample size computation and any required assumptions
- If no explicit power analysis was used, you should describe how you decided what sample (replicate) size (number) to use

Please outline where this information can be found within the submission (e.g., sections or figure legends), or explain why this information doesn’t apply to your submission:

Sample sizes were not determined in advance but rather depended on the size of the observed effects as well as on the reproducibility of a given experiment. The effects reported, in cell experiments and in biochemical reconstitutions, are very large within one set of experiments (with the same cell culture or the same protein prep). To get a robust conclusion, the experiment was then repeated (as indicated in the figure legends) with different cells, or different protein / lipid preparations.

**Replicates**

- You should report how often each experiment was performed
- You should include a definition of biological versus technical replication
- The data obtained should be provided and sufficient information should be provided to indicate the number of independent biological and/or technical replicates
- If you encountered any outliers, you should describe how these were handled
- Criteria for exclusion/inclusion of data should be clearly stated
- High-throughput sequence data should be uploaded before submission, with a private link for reviewers provided (these are available from both GEO and ArrayExpress)

Please outline where this information can be found within the submission (e.g., sections or figure legends), or explain why this information doesn’t apply to your submission:

The information on the number of independent experiments is given in the figure legends. In the case of cellular experiments, an independent experiment means that the cultures were grown at a different time from a new starting culture. In the case of biochemical experiments with purified proteins, independent experiments were likewise performed at different times. We used at least two different batches of purified protein stocks for each construct, as well as different batches of lipids (ordered from the same supplier).

All experimental data are shown and included in the figures; no data points were excluded from analysis.

**Statistical reporting**

- Statistical analysis methods should be described and justified
- Raw data should be presented in figures whenever informative to do so (typically when N per group is less than 10)
- For each experiment, you should identify the statistical tests used, exact values of N, definitions of center, methods of multiple test correction, and dispersion and precision measures (e.g., mean, median, SD, SEM, confidence intervals; and, for the major substantive results, a measure of effect size (e.g., Pearson's r, Cohen's d)
- Report exact p-values wherever possible alongside the summary statistics and 95% confidence intervals. These should be reported for all key questions and not only when the p-value is less than 0.05.

Please outline where this information can be found within the submission (e.g., sections or figure legends), or explain why this information doesn’t apply to your submission:

Following the suggestion in a recent paper in J Cell Biol (Lord et al., 2020), we aimed, as much as possible, to show all data points in our graphs. Where appropriate, we used ‘super-plots’ to represent at the same time data points obtained in independent experiments. Elsewhere, data are plotted as mean +/- SD, as indicated in figure legends.

(For large datasets, or papers with a very large number of statistical tests, you may upload a single table file with tests, Ns, etc., with reference to sections in the manuscript.)

**Group allocation**

- Indicate how samples were allocated into experimental groups (in the case of clinical studies, please specify allocation to treatment method); if randomization was used, please also state if restricted randomization was applied
- Indicate if masking was used during group allocation, data collection and/or data analysis

Please outline where this information can be found within the submission (e.g., sections or figure legends), or explain why this information doesn’t apply to your submission:

Not applicable

**Additional data files (“source data”)**

- We encourage you to upload relevant additional data files, such as numerical data that are represented as a graph in a figure, or as a summary table
- Where provided, these should be in the most useful format, and they can be uploaded as “Source data” files linked to a main figure or table
- Include model definition files including the full list of parameters used
- Include code used for data analysis (e.g., R, MatLab)
- Avoid stating that data files are “available upon request”

Please indicate the figures or tables for which source data files have been provided:

We provide source data for figures: 2, 3D, 3E, 4A-D, 5D, 5F, 6C, 6E, 6G, 6H, 7B, 7D, 8B, 8C (all figures with numerical data).
